# Cu_*n*_ Clusters (*n* = 13, 43, and 55) as Possible Degradant Agents of *m*SF_6_ Molecules (*m* = 1, 2): A DFT Study

**DOI:** 10.1021/acsomega.2c04020

**Published:** 2022-09-14

**Authors:** S. Mejía Sintillo, Alejandro Bautista Hernández, Alejandra Alicia Peláez Cid, Wilfredo Ibarra Hernández, M. Salazar Villanueva

**Affiliations:** † Benemérita Universidad Autónoma de Puebla, Facultad de Ingeniería, Apdo. Postal J-39, Puebla, 72570, Mexico; ‡ CIICAP UAEM, Avenida Universidad 1001, Chamilpa, 62209 Cuernavaca, Morelos, 62209, México

## Abstract

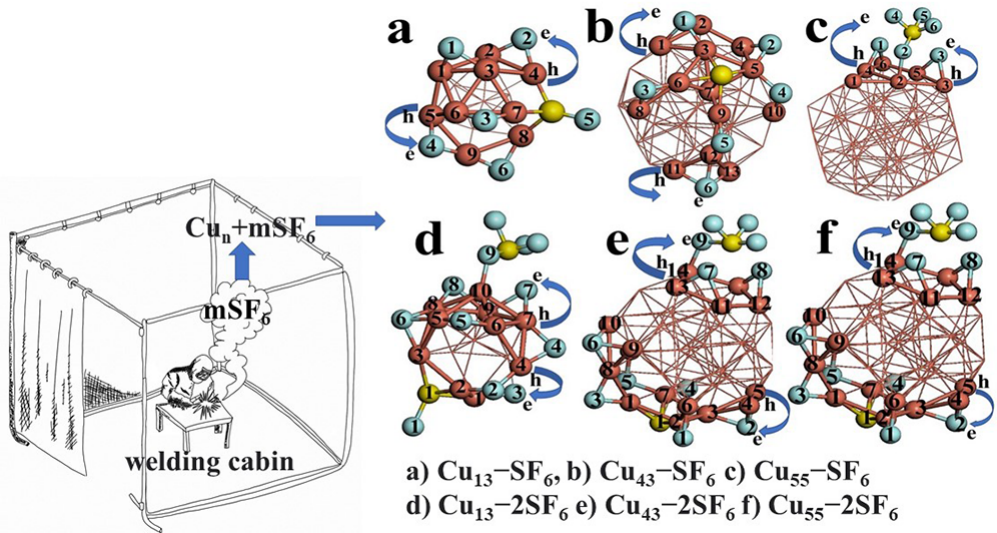

In order to obtain the structural and electronic properties of pristine copper clusters and Cu_13_–SF_6_, Cu_43_–SF_6_, Cu_55_–SF_6_, Cu_13_–2SF_6_, Cu_43_–2SF_6_, and Cu_55_–2SF_6_ systems, DFT calculations were carried out. For Cu_13_–*m*SF_6_, its surface suffers a drastic deformation, and Cu_43_–*m*SF_6_ at its outer surface reveals strong interaction for the first chemical molecule; when the second molecule is interacting, these outer surfaces are not severely affected. These two cases degraded fully the first SF_6_ molecule; however the second molecule is bonded to the latter systems and for Cu_55_–*m*SF_6_ (*m* = 1 and 2) a structural transformation from SF_6_ →SF_4_ appears as well as inner and outer shells that display slight deformations. The electronic gaps do not exhibit drastic changes after adsorption of *m*SF_6_ molecules, and the magnetic moment remains without alterations. The whole system shows thermal and vibrational stability. In addition, for Cu_13_–*m*SF_6_ the values of the optical gap and intensity of the optical exhibit changes with respect to the pristine case (Cu_13_), and the rest of the systems do not exhibit major oscillations. These icosahedral copper clusters emerge as a good option to degrade *m*SF_6_ molecules.

## 1 Introduction

SF_6_ (sulfur hexafluoride) is a greenhouse gas that is widely used in industrial processes as an insulating medium for electrical gas-insulated equipment,^1^ and due to its high chemical stability, the residence time in the atmosphere could be 3200 years.^2^ Several efforts have been devoted to finding alternatives to reduce or sense this kind of emissions.^3^ However, despite these attempts, it is desirable to find new alternatives to decrease this emission of greenhouse gas. Therefore, it is very important to sense, adsorb, or degrade this SF_6_ molecule toward minor subproducts.^4−7^ The sensing mechanism is governed on the change of electrical resistance of the so-prepared device caused by the interaction between the target gas and the nanomaterials;^8,9^ thus, Iwabuchi et al.^10^ adopted semiconducting ZnO thin films to realize the detection of several gas species successfully. 2D structures such as graphene and phosphorene have been studied to adsorb this SF_6_ molecule, and a physisorption effect was found.^11−13^ It should be mentioned that MoS_2_ and phosphorene have recently gained immense interest for gas sensing applications, which have also been theoretically reported.^14,15^ Despite the fact that single-walled and multiwalled CNT as well as graphene were all proposed for sensing SF_6_-decomposed species,^16−18^ the bonding governed by weak van der Waals interactions among these chemical species is not suitable for sensing molecules. However, this low interaction was overcome by means of embedding or doping impurity atoms such as transition metals^19−24^ and nonmetals,^25^ principally. On the other hand, graphene/metal oxide composites have been raised as advanced materials to capture diverse harmful gases such as HgCl_2_ and CO_2_. Mananghaya et al.^26^ have proposed graphene/CaO nanocomposite to understand the effects of temperature on the adsorption ability of the above chemical complexes by means of DFT/B3LYP calculations. Thus, porphyrin is a novel nanomaterial that exhibits covalent organic frameworks, and this has been used to adsorb greenhouse gases such as CO, CO_2_, and CH_4_ using DFT calculations by Suresh et al. successfully.^27^ Doped fullerenes with an externally oriented electric field have used for this goal. The doping site can control the structural, electronic, and energetic characteristics of the C_19_Si system, and as consequence, to adsorb these harmful gases, the DFT calculations were performed with different hybrid functionals.^28^ Nevertheless, metallic clusters have still not been considered to adsorb this kind of molecule, but due to their unique physical and chemical properties these are a good option, such as copper clusters. On the other hand, metallic clusters have shown good properties as sensors and for storage and degradation of some harmful gases.^29−31^ Several studies based on DFT and Monte Carlo dynamics have been performed to find the structural and electronic properties of copper clusters.^32−34^ All of them exhibit icosahedral geometry as the ground state; however, a transition from icosahedral → decahedral was found by Kabir et al. for the Cu_43_ cluster.^32^ The high chemical reactivity of these metallic clusters makes them potential vehicles for degradation/adsorption of the SF_6_ molecule. Previously, a theoretical study by means of molecular dynamics simulation revealed the critical temperature at which SF_6_ is degraded to derive complexes such as SF_2_, SF_3_, and SF_4_, respectively.^35^ This last study is analyzed because it is relevant to know if some of these subproducts are found in the present work. Therefore, it is necessary to perform a deep study about chemical interactions among icosahedral copper clusters, such as Cu_13_, Cu_43_, and Cu_55_, and SF_6_ molecules to follow the structural and electronic changes versus cluster size. This work is organized in the following way: section 2, parameters of calculation are detailed, section 3, pristine and SF_6_ bonded clusters are discussed, and section 4, some conclusions are drawn.

## 2 Computational Methodology

All systems analyzed in this work were performed using the density functional theory (DFT)^36^ approach as implemented in the DMol3 software,^37,38^ for both cases. The generalized gradient approximation has been chosen to describe the exchange–correlation interaction employing the Perdew–Burke–Ernzerhof (PBE) expression.^39^ To account for van der Waals forces we used a full optimization of the whole system with the Tkatchenko–Scheffler (TS) scheme due to its ability to describe large long range interactions.^40^ The use of this correction reduces the over/underestimation of adsorption energy values calculated for these chemical species onto titanium dioxide clusters. We have selected a basis set composed of a double numerical basis (4s and 3d) with polarized function (4p), and an all-electron calculation has been considered. The convergence criterion of optimization was set to 1 × 10^–5^ eV Å^–1^ for the energy gradient and 5 × 10^–4^ Å for the atomic displacements. The charge density is converged up to 1 × 10^–6^, which allows a total energy convergence of 1 × 10^–5^ eV. In the generation of the numerical basis sets, a global orbital cutoff of 5.2 Å was used. All calculations were carried out without spin restrictions, which allow for establishing the lowest energy geometries. The condition of noncomplex frequencies was established as the stability criterion for the studied systems. The electronic gaps (*E*_g_) were evaluated for the lowest energy structures from their corresponding energy differences between the HOMO and LUMO. It is well-known that values of *E*_g_ are underestimated by using DFT calculations; however, in Table 1 our results are compared with other works, and they agree. The optical properties were performed by the PBE0 hybrid functional,^41^ all of the systems in the ground state were placed in a box of 30 × 30 × 30 Å to avoid some possible interaction between these systems, and then one energy point was calculated to obtain the absorption spectra and, from them, the values of optics gap. In order to evaluate the thermal stability of the whole set of systems studied, ab initio molecular dynamics were performed at 300 K with steps of 1 fs during 1 ns to obtain their potential energy surface (PES) as well as to follow any possible structural change due to exposition at room temperature. The PES plots were obtained at the PBE functional/double numerical plus d-functions, this theory level was chosen due to the heavy computational cost; however, the results obtained are considered high enough quality to describe the thermal stability of these systems analyzed. The binding energy is calculated as *E*_b_/atom = (*X*_*n*_ – *nX*)/*n* where *X*_*n*_ is the total energy of the system (Cu_*n*_ or SF_6_), *X* is the sum of the energies of individual atoms, and “*n*” is the number of atoms for pristine cases. The adsorption energy was evaluated by means of the following equation
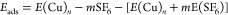
where *E*(Cu)_*n*_–SF_6_, *E*(Cu)_*n*_, and *E*(SF_6_) are the total energy of *m*SF_6_ molecules attached to the metallic cluster, metallic cluster bare, and *m*SF_6_ molecules isolated, respectively.

**Table 1:** Binding Energy Per Atom (*E*_b_/atom) and Electronic Gap Energy (*E*_g_) (eV)^a^

	*E*_b_/atom			*E*_g_		ABL		*l*_1_	*l*_2_
S	*a*	*b*	*c*	*a*	*b*	*a*	*d*	*a*	*a*
SF_6_	3.28			6.01				3.23	
Cu_13_	2.15	2.43	2.30	0.10	0.15	2.54	2.575	4.89	
Cu_43_	2.61	2.97	2.85	0.04	0.25	2.58		4.93	7.14
Cu_55_	2.70	3.09	2.95	0.07	0.05	2.59		4.94	9.72

a The average bond length (ABL) as well as *l*_1_ and *l*_2_ lengths are in Å. S means each one of the systems shown. The labels *a*, *b*, *c*, and *d* are related to data of this work and refs (5), (6), and (7), respectively.

## 3 Results and Discussion

### 3.1 Structural, Electronic, and Magnetic Properties of Cu_13_, Cu_43_, and Cu_55_ Metallic Clusters and SF_6_ Complex

**Figure 1:**
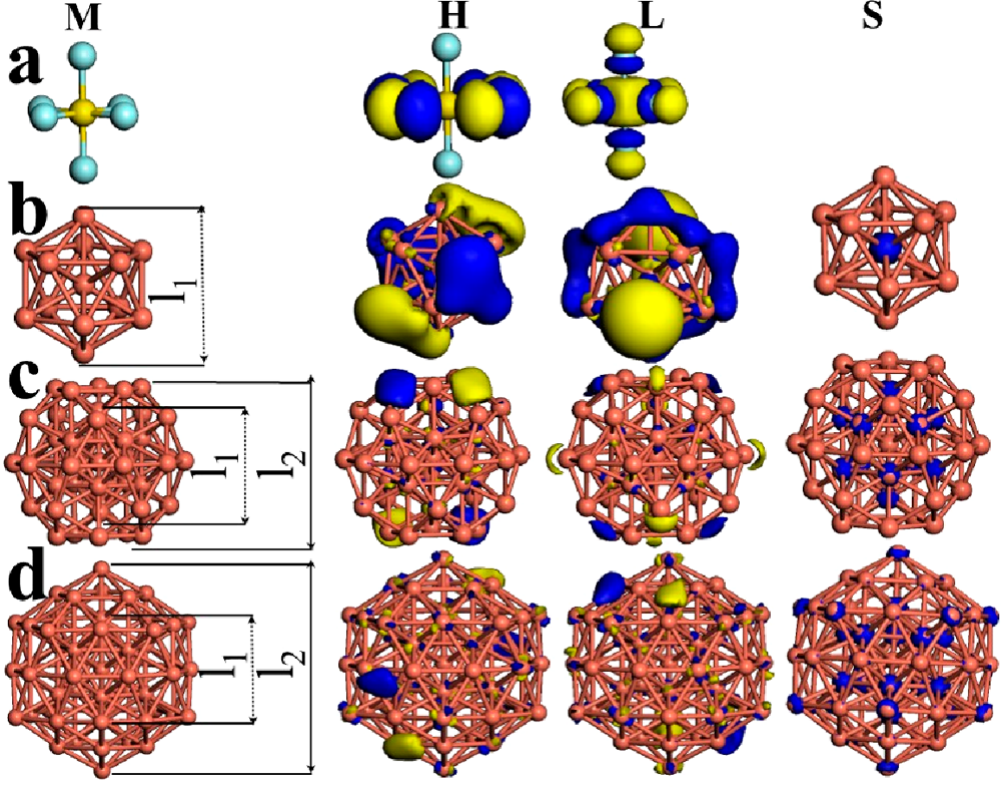
Models (M) for (a) SF_6_, (b) Cu_13_, (c) Cu_43_, and (d) Cu_55_ are displayed. The frontier orbitals HOMO (H) and LUMO (L) as well as spin density (S) are shown for these systems. The *l*_1_ and *1*_2_ labels mean the distance from end to end of core and shell of these metallic clusters, respectively. The iso-surfaces were plotted at 0.03 eV/Å^3^.

The SF_6_ exhibits symmetry like the 6-fold coordinated molecule (*O*_*h*_); thus, the sulfur atom is located at the center and it is surrounded by six atoms of fluorine, where four atoms form a regular square in a horizontal way and the other two atoms are perpendicular to this plane (see Figure 1a). The value of S–F bond is 1.62 Å, and this is the same for the six bonds associated to this chemical specie. The angle formed by F–S–F is 90° for all cases; therefore, this is a very symmetric molecule and it has been proved to be stable at room temperature. The binding energy per atom (*E*_b_/atom) confirms the above statement because this value is 3.28 eV. The *l*_1_ distance value is 3.23 Å, indicating that *l*_1_ for SF_6_ is shorter than the whole set of metallic clusters considered in this work; thus, it is possible that a strong interaction exists among these systems.

Thus, because of its electronic properties, the HOMO iso-surface for SF_6_ is located on four atoms of fluorine with σ bonds whereas LUMO displays π*−π* stacking on six of its fluorines as well as around the sulfur atom; thus, these effects generate an electronic behavior like insulator with a electronic gap value of 6.01 eV. This information is corroborated by means of partial density of states (PDOS) plot depicted in Figure 1S (Supporting Information) because of the smooth contribution of “s” electrons, and practically all “p” electrons show major participation for the SF_6_ molecule. The ground state is found at *M* = 1; therefore, a magnetic behavior is not displayed. On the other hand, the Cu_55_ cluster is a regular icosahedron (*I*_*h*_ symmetry), and this is formed by two icosahedrons, one of them of 13 atoms and the other one of 42 atoms, and they work as the “core” and “shell”, respectively. The Cu_43_ cluster is an icosahedron too, and it is formed by one icosahedron of 13 atom (core) and 30 atoms around of it (shell) to give the final geometry. The Cu_13_ cluster shows icosahedral disposition, as displayed in Figure 1b, c, and d, respectively. On the other hand, the values of the *E*_b_/atom exhibit an increasing tendency for Cu_*n*_ (*n* = 13, 43, and 55 atoms); hence, there is an energetic difference (Δ*E*) of 0.46 and 0.09 eV between *n* = 13 and 43 and *n* = 43 and 55, respectively. This structural fact indicates high stability and convergence toward the bulk regime for these metallic clusters. The value distance *l*_1_ (inner shell) for the three cases practically remains the same; however, those values associated with the outer shell (*l*_2_) suffer an increase of about 2.58 Å between *n* = 43 and 55 systems. This result could improve the chemical interaction with SF_6_ molecule. Thus, focusing on their electronic properties, the HOMO electronic distribution for the Cu_13_ cluster is shown on the bonds of the outer shell and LUMO is concentered like one ring and on the atoms at top and down; however, for Cu_43_ and Cu_55_, HOMO is located on the outer shell and with minor participation on the core, whereas LUMO is distributed in similar way in both cases, as shown in Figure 1b–d. The values of *E*_g_ reveal that these pristine copper clusters possess an electronic behavior like metal, and this tendency increases with size. The energetic minimum for these three copper clusters is found at *M* = 2; furthermore, they have 1 μ_B_ associated to each one. The spin density for Cu_13_ is located at the center; however, for Cu_43_ this is distributed on the atoms of the central icosahedron (core) and for Cu_55_ shows the same behavior but 12 atoms of outer shell exhibit this distribution as well. Thus, partially summarizing, from Figure 1S, for *n* = 13 and 43, there are is considerable participation by “s” and “p” electrons; however, for *n* = 55, this contribution is reduced drastically and “d” electrons government the electronic behavior of this metallic cluster. Thereby, it is expected that clusters with *n* = 13 and 43 are bonded more tightly with this chemical species than for *n* = 55. The latter fact is analyzed and discussed in the next section.

### 3.2 Structural, Vibrational, and Thermal Properties of the Cu_n_–*m*SF_6_ (*n* = 13, 43, and 55) (*m* = 1, 2) Systems

**Figure 2:**
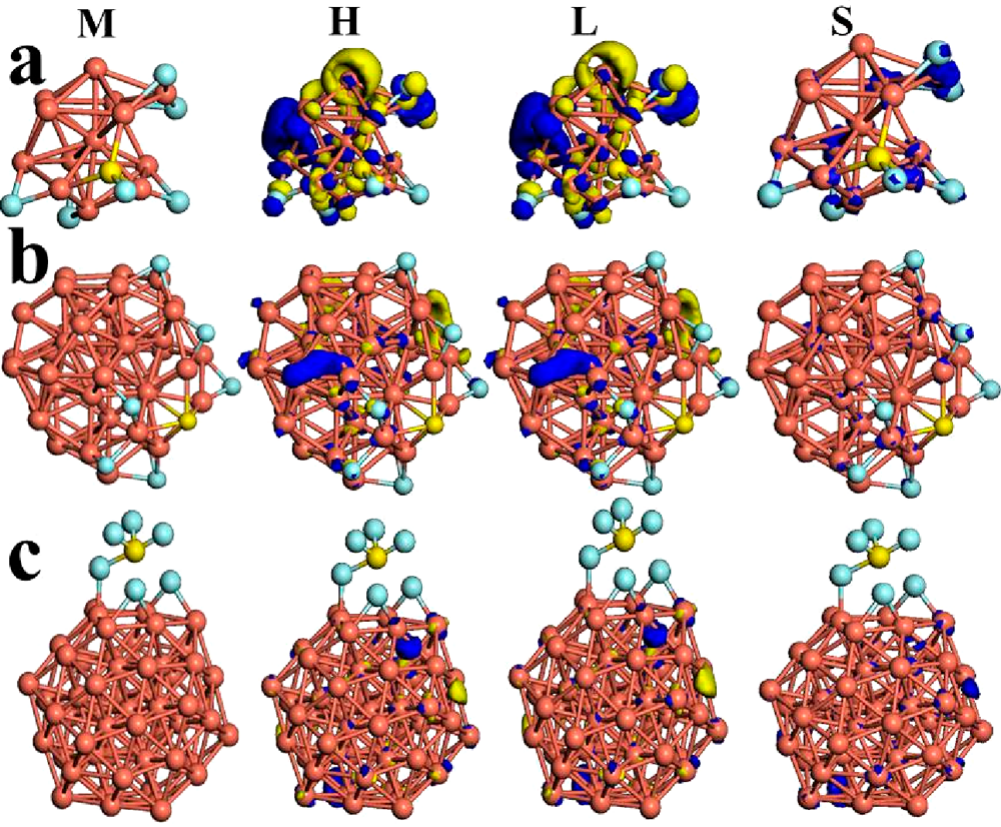
Models (M) for (a) Cu_13–_SF_6_, (b), Cu_43_–SF_6_, and (c) Cu_55–_SF_6_ are displayed. The frontier orbitals HOMO (H) and LUMO (L) as well as spin density (S) are shown for these systems. The iso-surfaces were plotted at 0.03 eV/Å^3^.

**Figure 3:**
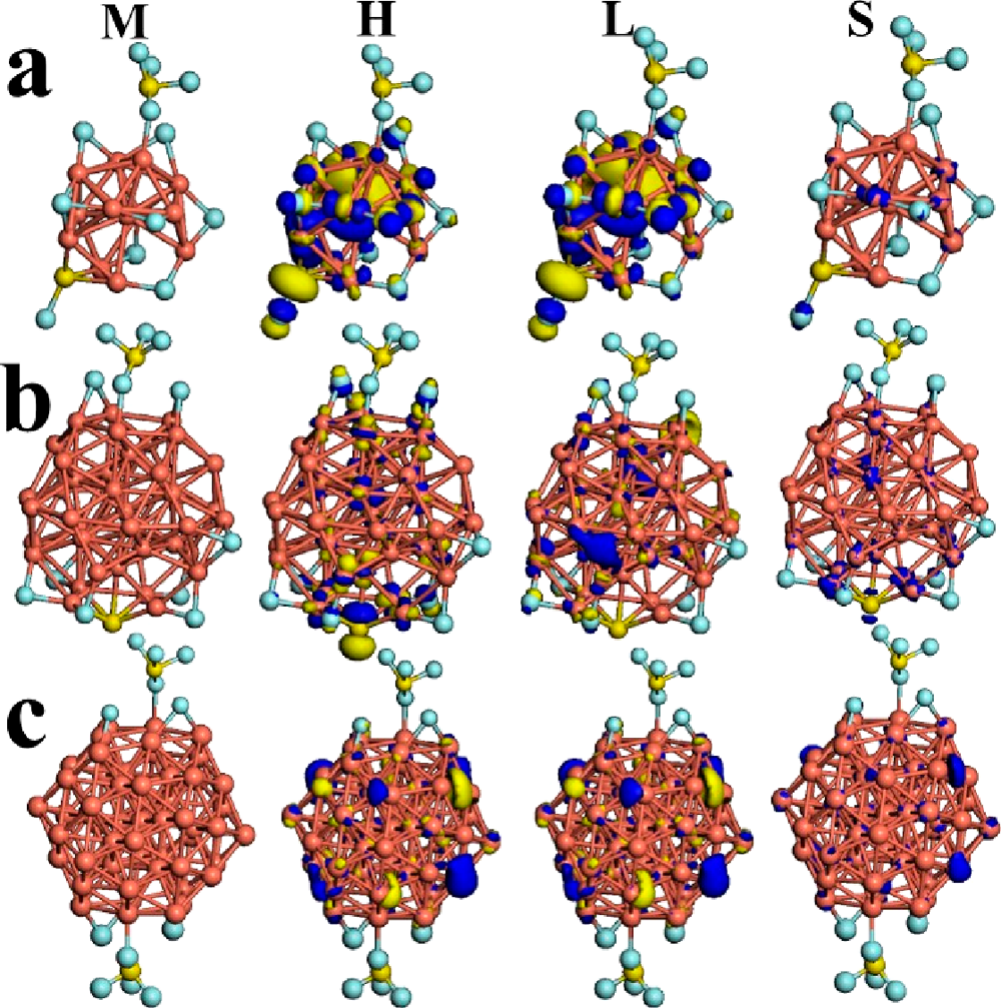
Models (M) for (a) Cu_13_–2SF_6_, (b) Cu_43_–2SF_6_, and (c) Cu_55_–2SF_6_ are displayed. The frontier orbitals HOMO (H) and LUMO (L) as well as spin density (S) are shown for these systems. The iso-surfaces were plotted at 0.03 eV/Å^3^.

Due to the high symmetry of metallic icosahedral clusters, there are many equivalent adsorption sites for the SF_6_ complex onto Cu_13_, Cu_43_, and Cu_55_ systems; in this work, one triangular face was chosen as the initial geometry and its opposite end for *m* = 1, 2, respectively. Thus, for the Cu_13_–SF_6_ system, at the top site two atoms of fluorine are bonded to the metallic cluster, and at the bottom site, three atoms of fluorine are bonded with copper atoms in the sequence Cu–F–Cu and the sulfur atom is bonded to last fluorine atom, as depicted in Figure 2a. This interaction generates the degradation of the SF_6_ molecule, whereas the metallic cluster is deformed; as a result, a chemisorption effect is shown according to the adsorption value obtained (−9.89 eV). However, for the Cu_13_–2SF_6_ system, the first SF_6_ molecule remains without drastic structural changes and the second one is degraded to two Cu–F–Cu bonds plus one subproduct as SF_4_ bonded to one copper atom, as depicted in Figure 3a.

This subproduct agrees with the report of by Liu et al.^32^ and is recurrent in this investigation. This interaction smoothly decreases the value of adsorption energy (−9.72 eV). Despite of this minor variation, it is considered a strong adsorption between these chemical species and the bare cluster (Cu_13_). For the Cu_43_–SF_6_ system, the six fluorine atoms exhibit bonds like Cu–F–Cu, and the sulfur atom is bonded on one pentagonal face of the outer shell of the metallic cluster. The core (*l*_1_) does not participate in this very strong chemical interaction with a value of −11.28 eV (chemisorption), such as depicted in Figure 2b. On the other hand, for the Cu_55_–SF_6_ system after the full geometric optimization, two atoms of fluorine are bonded to the Cu_55_ cluster and SF_6_ suffers a chemical transition toward the SF_4_ subproduct; thus, this chemical complex is degraded due to this strong interaction (see Figure 2c). In Figure 2c we show in detail this triangular face (formed with six copper atoms) that works as an adsorption site; beside it is shown the SF_6_ molecule degraded for better visualization. This triangular face suffers a geometric distortion because two bonds are broken and two sides are stretched up around 7% with respect to the pristine cluster. The values of Cu–F bond length range from 2.08 to 2.13 Å; furthermore, this effect is associated with good stability.

**Figure 4:**
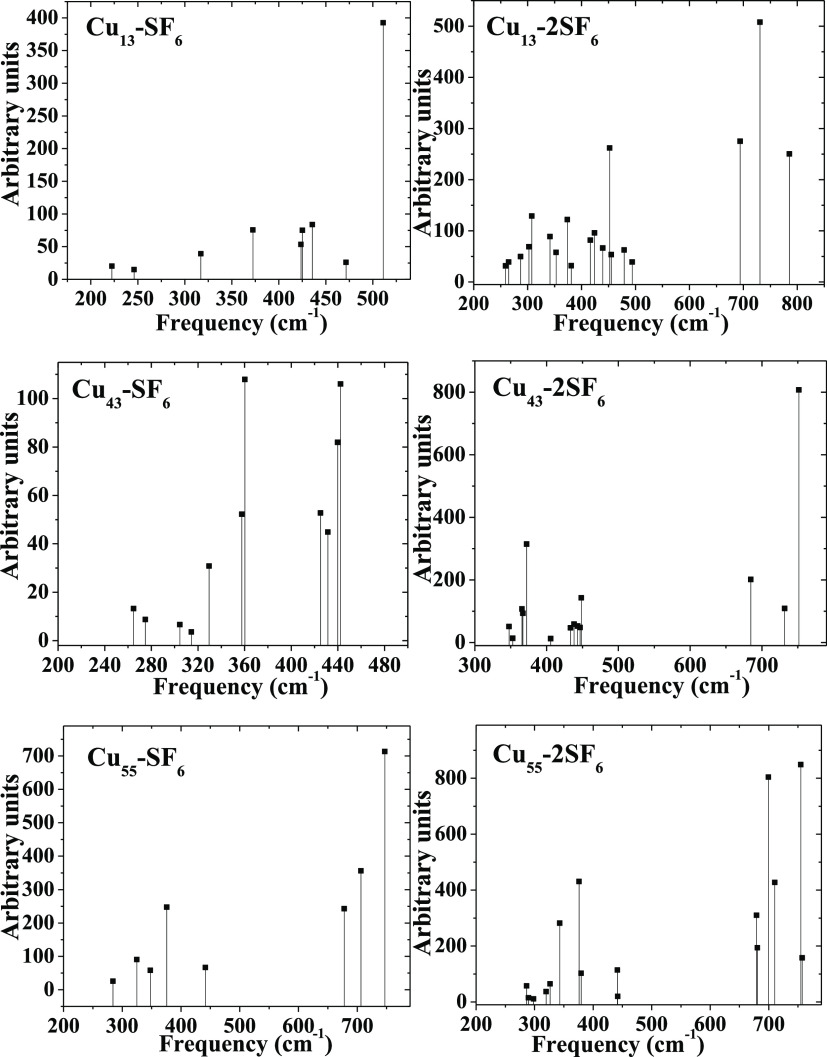
Vibrational spectra for the whole systems considered in this work are depicted, respectively.

Thus, for Cu_43_–2SF_6_ and Cu_55_–2SF_6_ systems the second chemical species exhibited similar structural behavior with respect to Cu_13_–2SF_6_; see Figure 3b and c, respectively. In particular, the second one has two subproducts that are geometrically opposite as mirrors (Figure 3c); thus, the adsorption values generated for Cu_43_–2SF_6_ and Cu_55_–2SF_6_ are −17.9 and −12.86 eV, respectively. From Figure 2S, it can be inferred that for *n* = 13 and *m* = 1 and 2 the values of adsorption energy change smoothly; nevertheless, for *n* ≥ 43 these values increases almost two times for *m* = 2 with respect to *m* = 1. This tight bonding is favored at *n* = 43; however, *n* = 55 can be synthesized with more abundance^29^ than *n* = 43. On the other hand, the vibrational modes are all real without imaginary contribution; hence, these three systems are stable, as shown in Figure 4. Thus, atoms of fluorine exhibit stretching modes from 316.1 up to 471.56 cm^–1^, whereas at 511.02, the most intensive peak, a similar mode is observed but this is caused by the F–S bond for Cu_13_–SF_6_. Stretching modes are associated with fluorine atoms at 264.83 cm^–1^, from 329.52 up to 362.83 cm^–1^, and a combination of stretching and blending modes are generated by fluorine atoms as well as from 425 up to 442.37 cm^–1^; there are stretching modes that come from fluorine plus sulfur atoms, respectively for Cu_43_–SF_6_. The Cu_55_–SF_6_ reveals a mixture of blending and stretching modes generated by fluorine atoms from 284.34 up to 375.61 cm^–1^, whereas at 441.47 cm^–1^ a blending mode associated with the SF_4_ subproduct is located and from 677.78 up to 746.91 cm^–1^ stretching modes are displayed for the last one. Nevertheless, for Cu_13_–2SF_6_, from 259.00 up to 352.78 cm^–1^ there are blending and stretching modes generated by Cu–F–Cu bonds and the SF_4_ subproduct as well as stretching modes at the opposite zone mentioned above from 416.15 up to 439.89 cm^–1^. A stretching mode is localized from 699.36 up to 785.47; this is generated by the SF_4_ subproduct. Thus, the Cu_43_–SF_6_ system exhibits stretching and wagging modes from 347.61 up to 405.60 cm^–1^ and stretching plus bending modes from 433.95 up to 751.65 cm^–1^ at the zone of the SF_4_ subproduct. Because Cu_55_–2SF_6_ displays similar adsorption compared to that with 3*m* = 1, from 286.00 up to 326.49 cm^–1^ stretching and blending modes are associated with both SF_4_ subproducts, besides from 379.61 up to 757.22 cm^–1^ stretching for the latter ones (see Figure 4).

**Figure 5:**
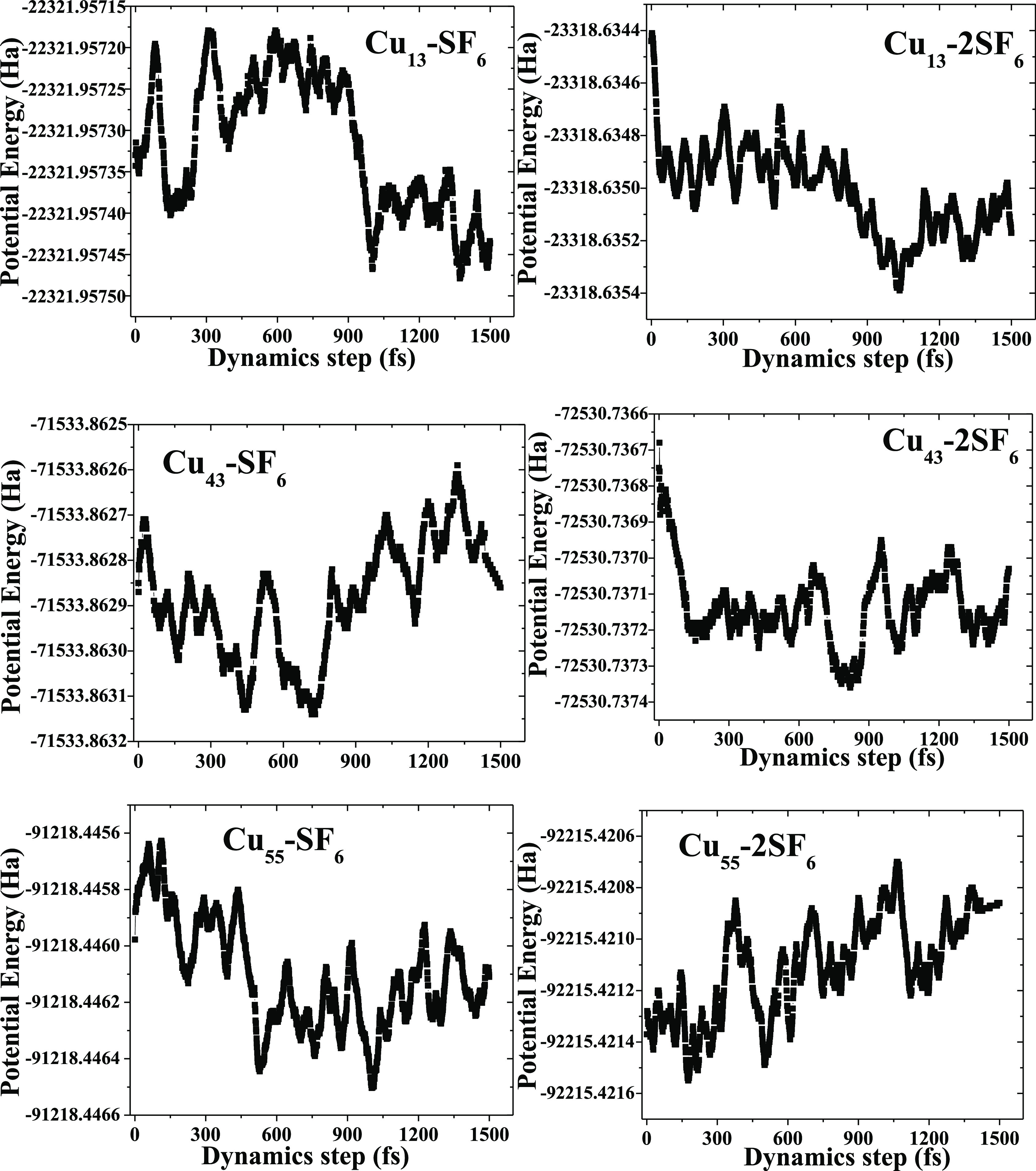
PES profiles for the whole set of systems considered in this work are depicted, from 0 to 1.5 ps.

In this sense, AIMD calculations at 300 K indicate thermal stability for the whole set of these systems; this is corroborated by means of EPS (energy potential surface) profiles, due to the maxim energetic difference values oscillate within the range of 0.01, 0.02, and 0.03 eV, for Cu_13_–SF_6_, Cu_43_–SF_6_, and Cu_55_–SF_6_ systems, respectively (see Figure 5).

**Figure 6:**
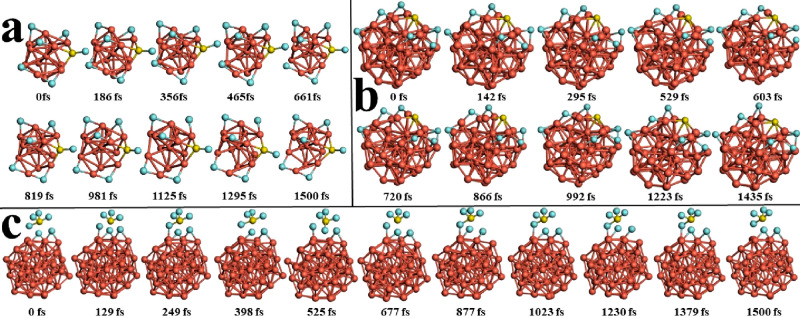
Snapshots of molecular dynamics trajectories of (a) Cu_13_–SF_6_, (b) Cu_43_–SF_6_, and (c) Cu_55_–SF_6_ systems, respectively, at longer time scales (*T* = 300 K, with a time step of 1 fs).

**Figure 7:**
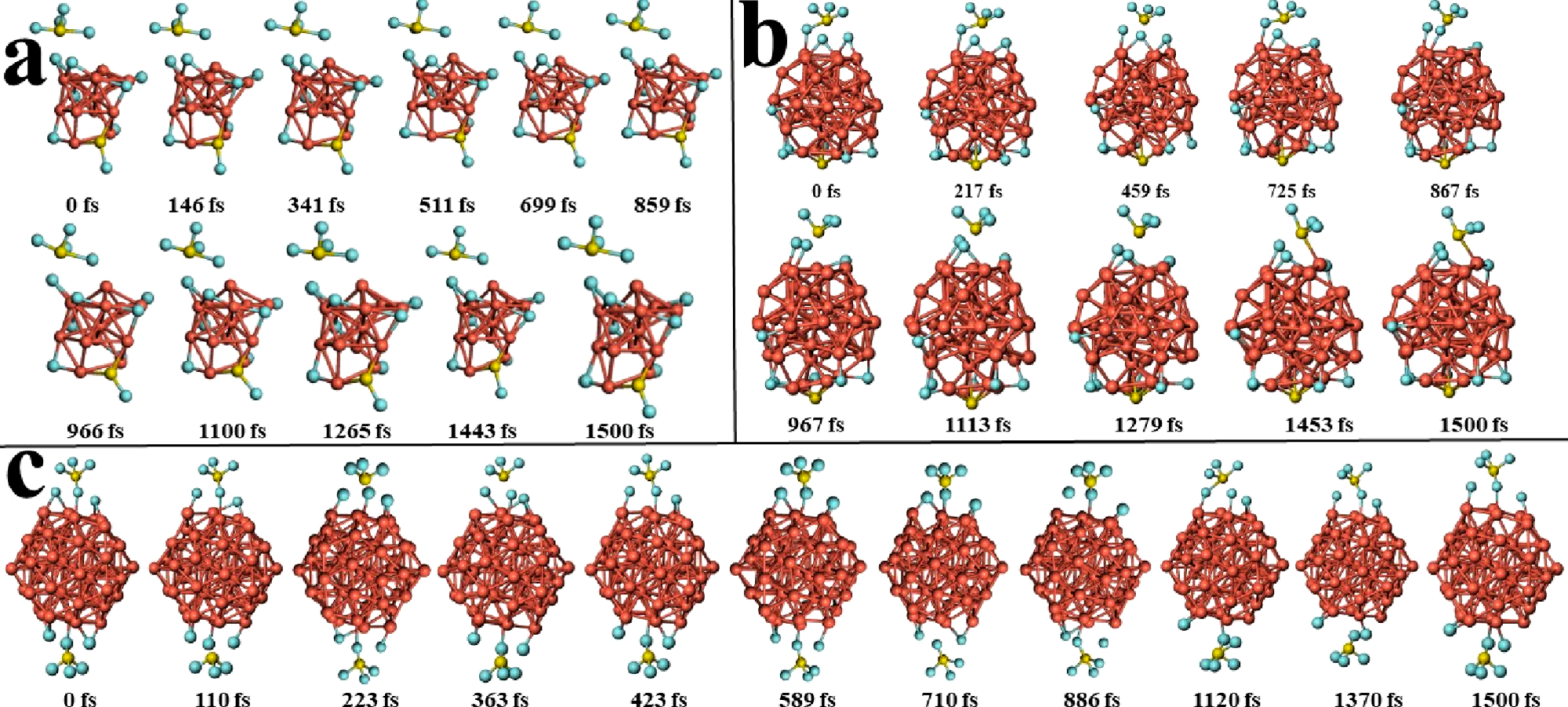
Snapshots of molecular dynamics trajectories of (a) Cu_13_–2SF_6_, (b) Cu_43_–2SF_6_, and (c) Cu_55_–2SF_6_ systems, respectively, at longer time scales (*T* = 300 K, with a time step of 1 fs).

From the above plots, it can be elucidated that these values fall in the range 0.03, 0.02, and 0.02 eV, respectively, for Cu_13_–2SF_6_, Cu_43_–2SF_6_, and Cu_55_–2SF_6_ systems. Thus, all of them are considered within low variation at room temperature and with good stability. In order to appreciate these structural variations at 300 K, the snapshots of Cu_n_–*m*SF_6_ (*n* = 13, 43, and 55) (*m* = 1, 2) systems are shown in Figures 6 and 7, respectively.

In each one, the Cu–F bonds are broken and then they are bonded again, and some Cu–Cu bonds suffer a similar effect, either in the inner (*l*_1_) or outer (*l*_2_) shell, for *n* = 43 and 55, both at *m* = 1 and 2. However, in the whole set of systems analyzed, the structures return to theirinitial configuration due to small energetic variations at this temperature.

### 3.3 Electronic, Magnetic, And Optical Properties of the Cu_n_–*m*SF_6_ (*n* = 13, 43, and 55) (*m* = 1,2) Systems

In this section, we explain the strong adsorption by means of electronic distribution of frontier orbitals (HOMO and LUMO), electronic transference (*Q*), partial density of states (PDOS), and shifts on optical spectra and dielectric function. Thus, for Cu_13_–SF_6_ its HOMO and LUMO electronic iso-surfaces are displayed on the part of no interaction of metallic clusters and on fluorine and copper atoms, principally (see Figure 2). The value of the electronic gap is increased to two times; however, it almost does not suffer changes (0.19 eV) with respect to the pristine case, and these values are displayed in Table 2.

**Table 2:** Values of Adsorption Energy (*E*_ads_), Electronic Gap (*E*_g_), and Optic Gap (*E*_o_) for systems listed are depicted^a^

system	Cu_13_	Cu_13_–SF_6_	Cu_13_–2SF_6_	Cu_43_	Cu_43_–SF_6_	Cu_43_–2SF_6_	Cu_55_	Cu_55_–SF_6_	Cu_55_–2SF_6_
*E*_g_	0.10	0.19	0.06	0.04	0.03	0.25	0.07	0.06	0.15
*E*_ads_		–9.89	–9.72		–11.18	–17.90		–6.84	–12.86
*E*_o_	0.80	0.30	0.95	0.05	0.01	0.06	0.01	0.01	0.01

a The unit used is eV for all values.

The spin density is located on the copper atoms and slightly on some fluorine atoms due to this strong chemical reaction obtained. Thus, Cu_43_–SF_6_ with its electronic distribution of HOMO and LUMO lies on copper atoms at no interaction zone, with smooth participation of fluorine atoms. The value of electronic gap remains with nearly of 0.03 eV and electronic behavior like-metallic still government this system (see Table 2). The spin density is located on copper atoms either on the outer or inner shell, respectively (see Figure 2d).

For Cu_55_–2SF_6_ the HOMO and LUMO iso-surfaces are located opposite to the adsorption site, and they are concentered on the bonds formed at outer shell (*l*_2_) with major participation of the “d” electrons; these are displayed in Figure 2c. The electronic behavior remains metallic (0.06 eV) as result of this chemical interaction.

**Figure 8:**
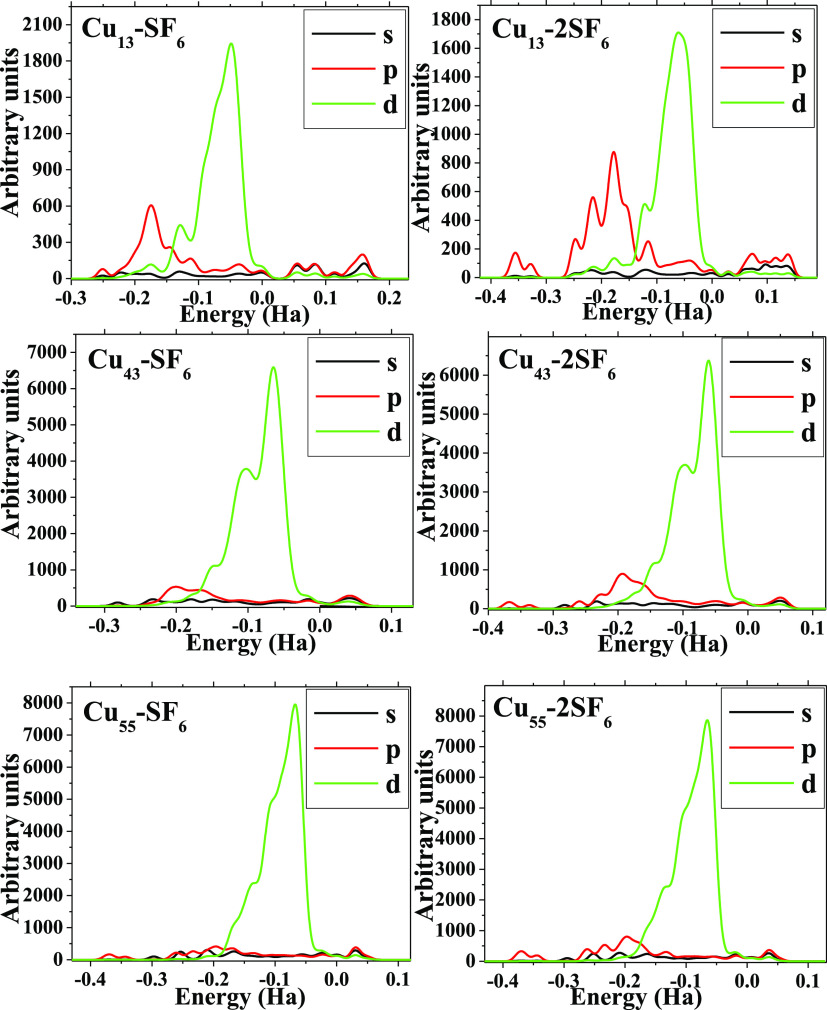
PDOS plots of each one of the systems considered in this work.

**Figure 9:**
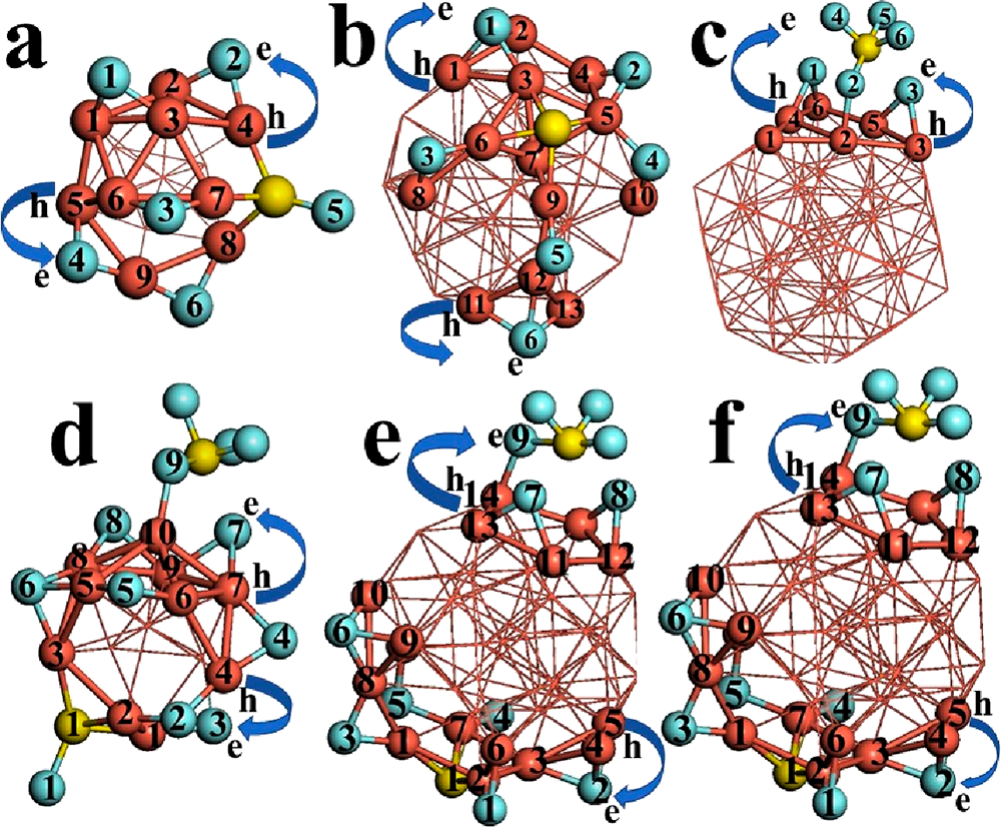
Models of (a) Cu_13_–SF_6_, (b) Cu_43_–SF_6_, (c) Cu_55_–SF_6_, (d) Cu_13_-2SF_6_, (e) Cu_43_-2SF_6_, and (f) Cu_55_–SF_6_ systems at the adsorption site are depicted. The atoms labeled are those that participate in adsorption, and the amount of the charge is listed in Table 1S (Supporting Information).

**Figure 10:**
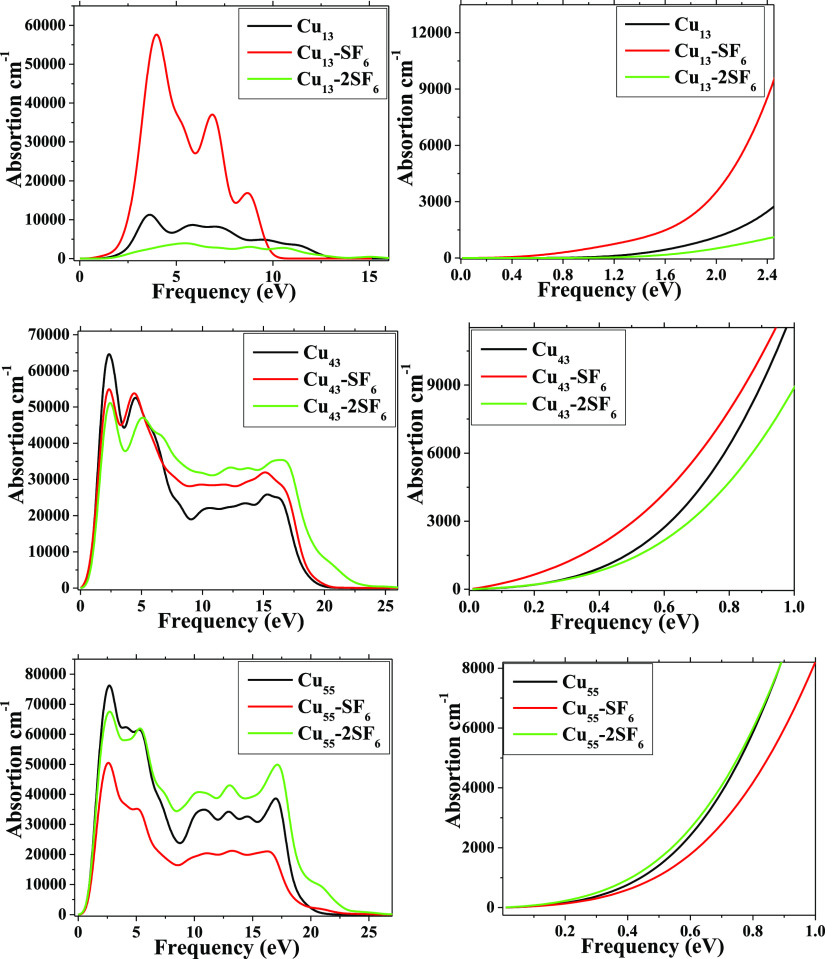
Absorption spectra of systems. (Right) Spectra depicted from 0 to 1.0 and 2.4 eV, respectively, for better appreciation.

Similar electronic behavior is found for *m* = 2. For Cu_13_–2SF_6_ its HOMO and LUMO electronic iso-surfaces are concentered at the central part of this system and around of S–F–3F bonds as well as the zone of the SF_4_ subproduct; these are distributed on two Cu–F–Cu bonds, as shown in Figure 3b. The spin density is located on copper atoms bonded to fluorine atoms at both ends. On the other hand, the HOMO and LUMO electronic distributions are located at both adsorption zones and over the inner shell of metallic cluster and spin density too. These homogeneous iso-surfaces explain the good stability and strong adsorption associated to Cu_43_–2SF_6_. Otherwise, Cu_55_–2SF_6_ their HOMO and LUMO are concentered at outer shell of metallic cluster and their spin density exhibits similar effect, as depicted in Figure 3c. Their electronic gap values do not exhibit drastic changes, and moreover, these systems retain electronic behavior like metallic (see Table 2). These features are corroborated by PDOS plots in Figure 8; the contributions of “p” electrons that come from SF_6_ molecule are decreasing as size cluster increases in both cases (*m* = 1 and 2), whereas “d” electrons do not suffer drastically changes (Cu_13_, Cu_43_, Cu_55_). These last ones lead to this electronic behavior for the systems studied in this work, and despite strong adsorption and structural distortions, the high chemical reactive of the metallic cluster prevails, hence allowing electronic behavior like-metal to remain after this strong interaction. From Table 1S of the Supporting Information it can be elucidated that the electronic charge (*Q*) migrates from copper atoms toward fluorine atoms among metallic clusters and SF_6_ molecule, at an adsorption site, for Cu_*n*_–*m*SF_6_ (*n* = 13, 43, and 55) (*m* = 1,2) systems. In Figure 9, the atoms at the adsorption site are labeled with respect to Table 1S. This effect is due to fluorine atoms possessing the highest electronic negativity, and the copper atoms with the [Ar]3d^10^4s^1^ electronic configuration tend to allow their electrons to form some kind of bond or tight interaction, as mentioned above. This fact leads to larger values of adsorption energy for these systems. In order to illustrate the way of optical properties change after adsorption process, the absorption spectra for Cu_n_–*m*SF_6_ (*n* = 13, 43, and 55) (*m* = 1, 2) systems are calculated and depicted in Figure 10. From there, the values of optical gap can be obtained as 0.8, 0.3, and 0.95 eV for Cu_13_, Cu_13_–SF_6_, and Cu_13_–2SF_6_, respectively. For *n* = 43 and 53 with *m* = 1 and 2, these values are around 0 eV, which are agrees with those of electronic gap values, as shown in Table 2.

This shift of these values is associated with the strong adsorption of the SF_6_ molecule on the metallic cluster as well as the participation of 2p electrons from fluorine atoms generate this important optical effect. Thus, the intensity of absorption spectrum for Cu_13_–SF_6_ is reduced by 4.5 times with respect to the pristine case; hence, the capacity of adsorption is reduced due to interaction with SF_6_ molecule. This effect is more located for *n* = 13 than the rest of the metallic clusters.

**Figure 11:**
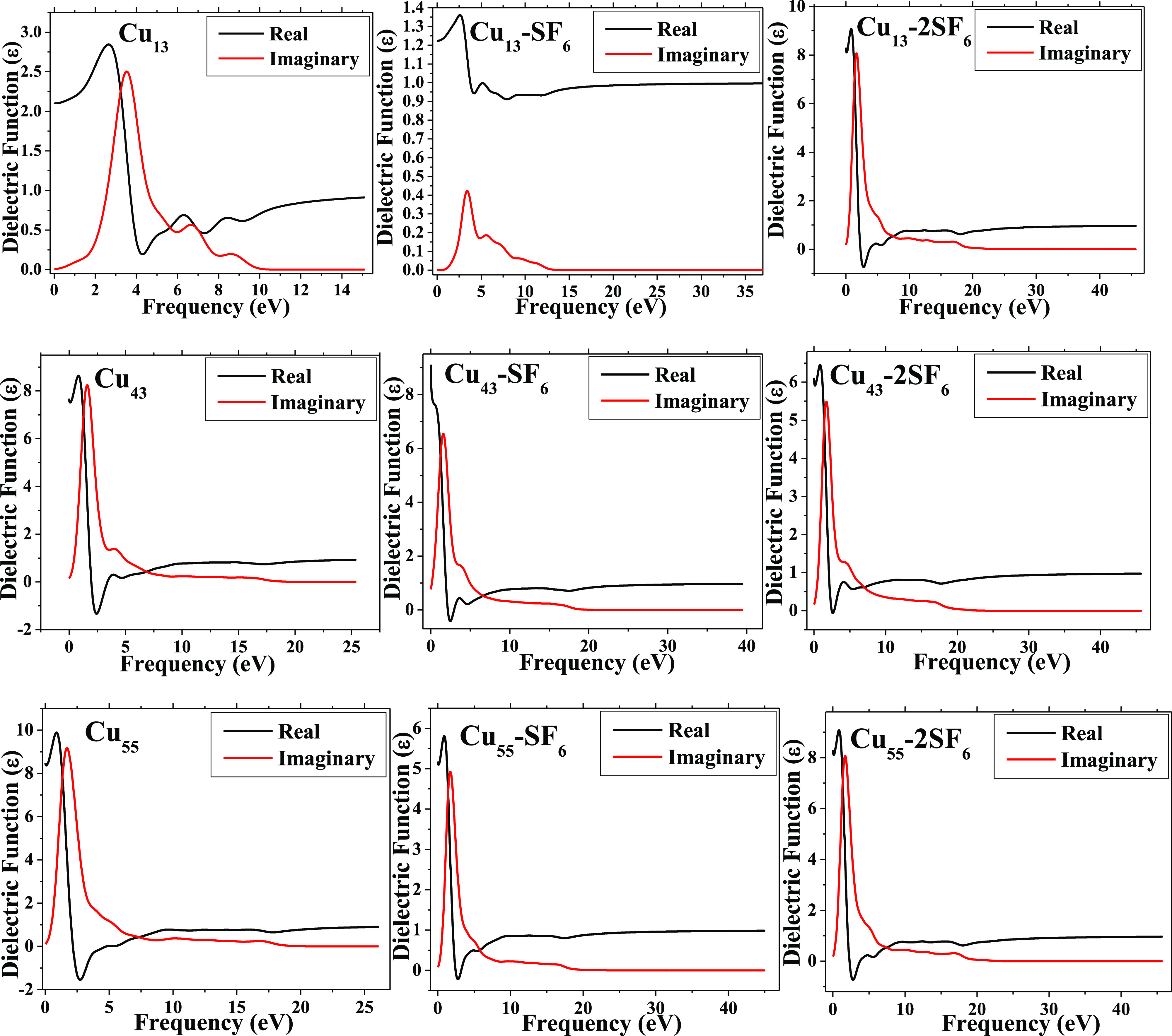
Plots of the dielectric function for each system are depicted, respectively.

In this sense, the dielectric function of the pristine cluster and cluster bonded to the SF_6_ molecule is affected due to the adsorption process, as depicted in Figure 11. Once more, the contribution of 2p electrons that comes from fluorine atoms leads to this optical process. The real part of the dielectric function at the frequency of 0 eV corresponds to the static dielectric constant; this value decreases from 2.2 eV (Cu_13_) up to 1.2 eV (Cu_13_–SF_6_) and increases up to 8.2 eV for Cu_13_–2SF_6_. Similar transitions are found for Cu_43_, Cu_13_–SF_6_, and Cu_13_–2SF_6_ with values of 7.5, 9.1, and 6.2 eV, respectively as well as for Cu_55_, Cu_55_–SF_6_ and Cu_55_–2SF_6_ with values of 8.2, 5.2, and 8.3 eV, respectively. Moreover, the adsorption of SF_6_ molecules on copper clusters is responsible of this important shift, as well. Therefore, from these two optical techniques can be observed when pristine metallic clusters have interacted with SF_6_ molecules in order to help experimentally recognize if the adsorption process has been performed successfully.

## 4 Conclusions

In order to perform the adsorption of *m*SF_6_ molecules (*m* = 1 and 2) onto pristine copper clusters (Cu_*n*_, *n* = 13, 43, and 55), DFT calculations were used for full geometrical optimization. There is a strong interaction among SF_6_ molecules and Cu_13_, Cu_43_, and Cu_55_ clusters; as a result, the outer surface (second shell) is drastically deformed. Hence, the surfaces in touch are strongly distorted for *n* ≤ 43 when the first molecule is bonded, and for the second they are partially degraded. However, for *n* = 55 the outer surface remains almost without variation, and it is found a structural transition from SF_6_ → SF_4_ in agreement with previously reported^8^ for *m* = 1 and 2. The electronic distribution of HOMO and LUMO is located on metallic clusters and some fluorine atoms (Cu_13_–SF_6_ and Cu_13_–2SF_6_), and the charge transference is from copper atoms toward fluorine atoms for the whole set of systems analyzed. According to these effects, the electronic behavior like-metallic remains. The spin density is displayed in an asymmetric way on copper atoms and some fluorine atoms; for *n* ≤ 43 and 55 this is concentered on the copper atoms, all of them with respect to pristine cases. The PES plots indicate great thermal stability at room temperature (300 K) in each one of the cases studied. The 2p electrons that come from the fluorine atoms of the SF_6_ complex lead to the decreasing tendency of intensity of the optical spectrum and gap as well as affect the dielectric function, respectively. These optical effects can be used on the experimental side to follow the adsorption process of these molecules. Thus, these copper clusters can be used to degrade them; nevertheless, for *n* ≤ 43 their surface area decreases because only part of them are free to interact with other molecules, while for *n* = 55 this is not distorted and it is a good candidate to be bonded with more molecules.

## Supporting Information Available

Plots of PDOS for pristine systems, adsorption energy (*E*_ads_) versus size (*n*) of metallic clusters, and the table of distribution of Mulliken charges at the adsorption site according to Figure 4 for Cu_*n*_–*m*SF_6_ clusters (*n* = 13, 43, and 55) (*m* = 1 and 2) (PDF)
