# Supplementary material for: Cun Clusters (n = 13, 43, and 55) as Possible Degradant Agents of mSF6 Molecules (m = 1, 2): A DFT Study
Source: ACS Omega. 2022 Sep 14;7(38):34401–11. doi: 10.1021/acsomega.2c04020 (PMC9521032; doi:10.1021/acsomega.2c04020)
Supplement: Supplementary file 1 — ao2c04020_si_001.pdf [file ao2c04020_si_001.pdf]

Cu_n clusters (n=13, 43 and 55) as possible degradant agents of mSF_6 molecules (m=1,2): A DFT study

S. Mejía Sintillo[1,2], Alejandro Bautista Hernández[1], Alejandra Alicia Peláez Cid[1], Wilfredo Ibarra Hernández[1] and M. Salazar Villanueva[1]*

[1]*Benemérita Universidad Autónoma de Puebla, Facultad de Ingeniería, Apdo. Postal J-39, Puebla, Pue., 72570, México.*

[2]*CIICAP UAEM, Avenida Universidad 1001, Chamilpa, 62209 Cuernavaca, Mor., 62209, México.*

*Corresponding author: martin.salazar@correo.buap.mx

Support information

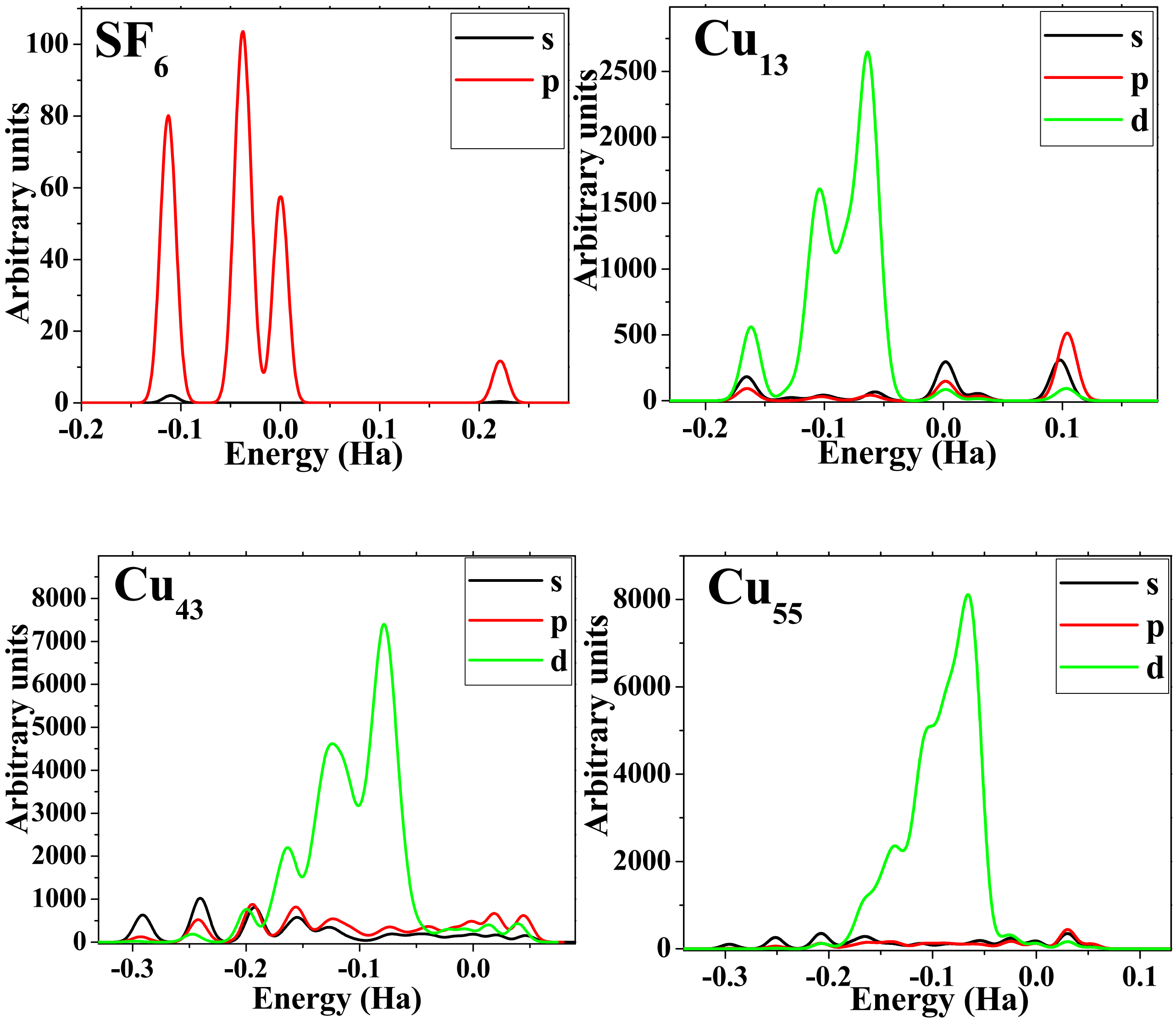

Figure 1S. The PDOS plots of pristine systems are depicted.

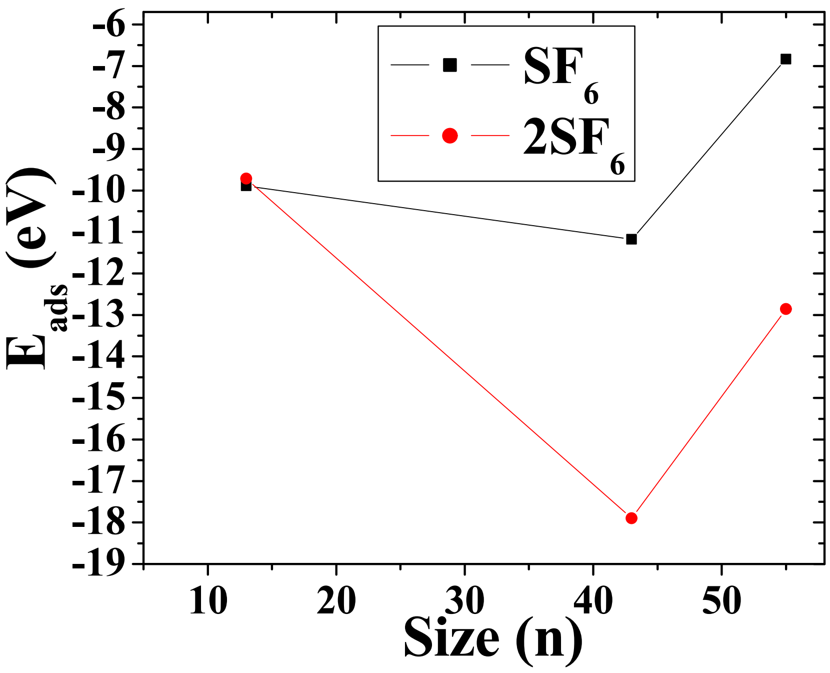

Figure 2S. The adsorption energy (E_{ads}) versus size (n) of metallic clusters is shown.

$Cu_{55}-SF_6$	Cu1	Cu2	Cu3	Cu4	Cu5	Cu6	F1	F2	F3	F4	F5	F6	S							
	0.09	0.09	0.04	0.10	0.08	0.09	-0.32	-0.26	-0.29	-0.12	-0.10	-0.16	0.53							
$Cu_{43}-SF_6$	Cu1	Cu2	Cu3	Cu4	Cu5	Cu6	Cu7	Cu8	Cu9	Cu10	Cu11	Cu12	Cu13	F1	F2	F3	F4	F5	F6	S
	0.11	0.10	0.16	0.16	0.04	0.22	0.18	0.01	0.13	0.19	0.184	0.10	0.10	-0.29	-0.32	-0.33	-0.31	-0.30	-0.29	-0.18
$Cu_{13}-SF_6$	Cu1	Cu2	Cu3	Cu4	Cu5	Cu6	Cu7	Cu8	Cu9	F1	F2	F3	F4	F 5	F 6	F7	S			
	0.10	0.13	0.20	0.19	0.11	0.21	0.19	0.17	0.26	-0.32	-0.31	-0.31	-0.28	-0.31	-0.24	-0.30	-0.05			
$Cu_{55}-2SF_6$	Cu1	Cu2	Cu3	Cu4	Cu5	Cu6	F1	F2	F3	F4	F5	F6	S1							
$Cu_{43}-2SF_6$	Cu1	Cu2	Cu3	Cu4	Cu5	Cu6	Cu7	Cu8	Cu9	Cu10	Cu11	Cu12	Cu13	Cu14	S1	F1	F2	F3	F4	F5
	0.34	0.32	0.26	0.16	0.18	0.45	0.35	0.17	0.17	0.18	0.17	0.18	0.20	0.18	-0.30	-0.51	-0.49	-0.51	-0.51	-0.52
	F6	F7	F8	F9																
	-0.49	-0.50	-0.49	-0.45																
$Cu_{13}-2SF_6$	Cu1	Cu2	Cu3	Cu4	Cu5	Cu6	Cu7	Cu8	Cu9	Cu10	S1	F1	F2	F3	F4	F5	F6	F7	F8	F9
	0.33	0.35	0.21	0.50	0.44	0.29	0.34	0.38	0.28	0.27	0.10	-0.37	-0.50	-0.51	-0.49	-0.50	-0.47	-0.47	-0.51	-0.44

Table 1S. The distribution of Mulliken charges at adsorption site according Figure 4 is presented for $Cu_{55}-SF_6$, $Cu_{43}-SF_6$, $Cu_{13}-SF_6$ systems. Cu, F, and S are labels related to cupper, fluorine and sulfur atoms, respectively.
